# Changes in patient-reported outcomes in patients with non-idiopathic pulmonary fibrosis fibrotic interstitial lung disease and progressive pulmonary fibrosis

**DOI:** 10.3389/fmed.2023.1067149

**Published:** 2023-06-30

**Authors:** Reoto Takei, Toshiaki Matsuda, Jun Fukihara, Hajime Sasano, Yasuhiko Yamano, Toshiki Yokoyama, Kensuke Kataoka, Tomoki Kimura, Atsushi Suzuki, Taiki Furukawa, Junya Fukuoka, Takeshi Johkoh, Yasuhiro Kondoh

**Affiliations:** ^1^Department of Respiratory Medicine and Allergy, Tosei General Hospital, Seto, Japan; ^2^Department of Respiratory Medicine, Nagoya University Graduate School of Medicine, Nagoya, Japan; ^3^Medical IT Center, Nagoya University Hospital, Nagoya, Japan; ^4^Department of Pathology, Nagasaki University Hospital, Nagasaki, Japan; ^5^Department of Radiology, Kansai Rosai Hospital, Amagasaki, Japan

**Keywords:** interstitial lung disease, progressive pulmonary fibrosis, progressive fibrosing interstitial lung disease, St George’s respiratory questionnaire, COPD assessment test, health-related quality of life, 6 min walk distance

## Abstract

**Background:**

Health-related quality of life (HRQoL) captures different aspects of the fibrotic interstitial lung disease (FILD) evaluation from the patient’s perspective. However, little is known about how HRQoL changes in patients with non-idiopathic pulmonary fibrosis (IPF) FILD, especially in those with progressive pulmonary fibrosis (PPF). The aim of this study is to clarify whether HRQoL deteriorates in patients with non-IPF FILD and to evaluate the differences in the changes in HRQoL between those with and without PPF.

**Methods:**

We collected data from consecutive patients with non-IPF FILD and compared annual changes in HRQoL over 2 years between patients with PPF and those without. The St George’s respiratory questionnaire (SGRQ) and COPD assessment test (CAT) were used to assess HRQoL. Changes in the SGRQ and CAT scores for 24 months from baseline were evaluated with a mixed-effect model for repeated measures.

**Results:**

A total of 396 patients with non-IPF FILD were reviewed. The median age was 65 years and 202 were male (51.0%). The median SGRQ and CAT scores were 29.6 and 11, respectively. Eighty-six (21.7%) showed PPF. Both SGRQ and CAT scores were significantly deteriorated in patients with PPF compared to those without PPF (*p* < 0.01 for both). Clinically important deterioration in the SGRQ and CAT scores were observed in 40.0 and 35.7% of patients with PPF and 11.7 and 16.7% of those without, respectively. PPF was significantly associated with clinically important deterioration in the SGRQ score (odds ratio 5.04; 95%CI, 2.61–9.76, *p* < 0.01) and CAT score (odds ratio 2.78; 95%CI, 1.27–6.06, *p* = 0.02).

**Conclusion:**

The SGRQ and CAT scores were significantly deteriorated in patients with non-IPF FILD and PPF. Considering an evaluation of HRQoL would be needed when assessing PPF.

## Introduction

Interstitial lung diseases (ILDs) are a large and heterogeneous group of lung disorders characterized by fibrosis and inflammation of the lung tissue. Various topics of ILDs including genetic variants or the utility of the International Classification of Functioning, Disability, and Health have been discussed and one of the recent hot topics of ILDs were disease progression (1–4). Idiopathic pulmonary fibrosis (IPF) is the symbolic and most frequent disease of fibrotic ILDs (FILDs) and IPF usually shows progression of fibrosis (1). Some FILDs other than IPF also have a progressive phenotype despite treatment (1, 5–10) and have been reported to show similar overall survival to IPF (5, 6). Recently, non-IPF FILDs with a progressive phenotype have been noted as a form of progressive pulmonary fibrosis (PPF) (1).

Studies have used variable definitions of a progressive phenotype, most of which cite a decline in pulmonary function, progression of radiological fibrosis and worsening of respiratory symptoms (1, 5–8, 11). Although these measures are useful to evaluate disease progression, they are not sufficient to assess patients’ feelings and functioning.

Health-related quality of life (HRQoL) captures different aspects of ILD from the patient’s perspective (12). Although deterioration of HRQoL lacks objectivity in practice, it has been thought to be highly meaningful for patients (13). However, little is known about whether HRQoL deteriorates in non-IPF FILD patients with PPF and whether there is a difference in the changes in HRQoL between those with and without PPF. We think investigating relationships between criteria for PPF and HRQoL lead to identifying PPF in terms of quality of life, shedding light on the significance of HRQoL, and revising the criteria for PPF. The aim of this study is to investigate whether there is a decline in HRQoL in patients with non-IPF FILD. Additionally, the study aims to evaluate and compare the differences in the changes of HRQoL between patients with and without PPF.

## Materials and methods

### Patient selection

The medical records of consecutive patients with non-IPF FILD who underwent initial evaluation at Tosei General Hospital (Seto, Japan) between January 2008 and July 2015 were retrospectively reviewed. We included patients with non-IPF FILD who had evaluated PPF based on our previous study (5). PPF, which had already been confirmed in the previous study (5), was defined as the presence of at least one of the following at 24 months from the initial evaluation: a relative decline in forced vital capacity (FVC) of at least 10%; a relative decline in FVC of ≥5–<10% with a relative decline in the diffusing capacity of the lung for carbon monoxide (DL_CO_) of at least 15%; a relative decline in FVC of ≥5–<10% with increased fibrosis on high-resolution computed tomography; and a relative decline in FVC of ≥5–<10% with progressive symptoms. The final diagnoses of non-IPF FILD were categorized as idiopathic non-specific interstitial pneumonia, fibrotic hypersensitivity pneumonitis, connective tissue disease-related ILD, idiopathic pleuroparenchymal fibroelastosis and unclassifiable ILD. Patients who died or underwent lung transplantation within 24 months from the initial evaluation were excluded.

### Study design

We collected data on HRQoL and exercise capacity at baseline, 1 year, and 2 years. We compared the annual changes in the HRQoL over 2 years between patients with and without PPF. The baseline data were collected at the initial evaluation of ILD.

The St George’s respiratory questionnaire (SGRQ) and COPD assessment test (CAT) were used to assess HRQoL. The SGRQ is a specific questionnaire for respiratory disease and provides three component scores for the domains of symptoms, activity, and impacts, as well as a total score (score range: from 0 to 100, with higher scores indicating greater impairment of HRQoL) (14). The CAT is composed of eight items related to symptoms of respiratory disease and their impact: cough, phlegm, chest tightness, breathlessness, activity limitation, confidence, sleep, and energy. Patients are asked to respond to all items using an identical 0–5 response scale (score range: from 0 to 40, with a score of 0 indicating no impairment) (15). Exercise capacity was evaluated using the 6 min walk test, according to the American Thoracic Society statement (16).

The minimal clinically important difference (MCID) was utilized to evaluate the deterioration of the SGRQ, CAT and the 6 min walk distance (6MWD) with the thresholds of 8 points, 5 points, 7 points, and 7 points for SGRQ symptom, activity, impact, and total scores, respectively (17); 4 points for CAT score (18); and 28 m for 6MWD (19). This study was carried out at a single hospital in compliance with the principles of the Declaration of Helsinki and approved by its institutional review board (IRB No. 1091, August 16th, 2022).

### Statistical analysis

The statistical tests used in this study were Fisher’s exact test and Mann–Whitney U test to compare categorical and continuous variables, respectively. A mixed-effect model for repeated measures was used to evaluate changes in SGRQ, CAT, and 6MWD over 24 months from baseline. A cumulative distribution function (CDF) plot was generated to visually present the relationship between HRQoL change scores or 6MWD change and PPF. The CDF plots used data from patients with and without PPF. All statistical tests were two-sided, and a significance level of *p* < 0.05 was used to determine statistical significance. The data were reported using descriptive statistics, such as mean, standard deviation, median, and interquartile range. Results of the statistical tests were reported with the corresponding *p*-values and confidence intervals (CI) when appropriate. Statistical analyses were performed using SPSS version 25.0 (SPSS Inc., Chicago, IL).

## Results

**Figure 1:**
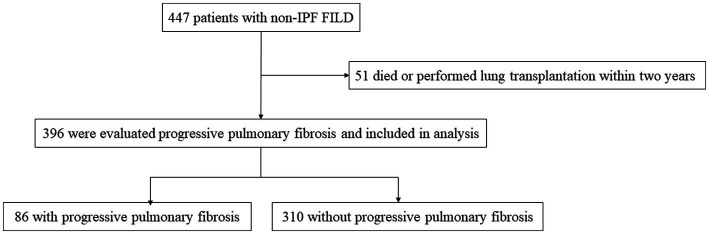
Screening and inclusion process for patients in the study. FILD, fibrotic interstitial lung disease; IPF, idiopathic pulmonary fibrosis.

**Figure 2:**
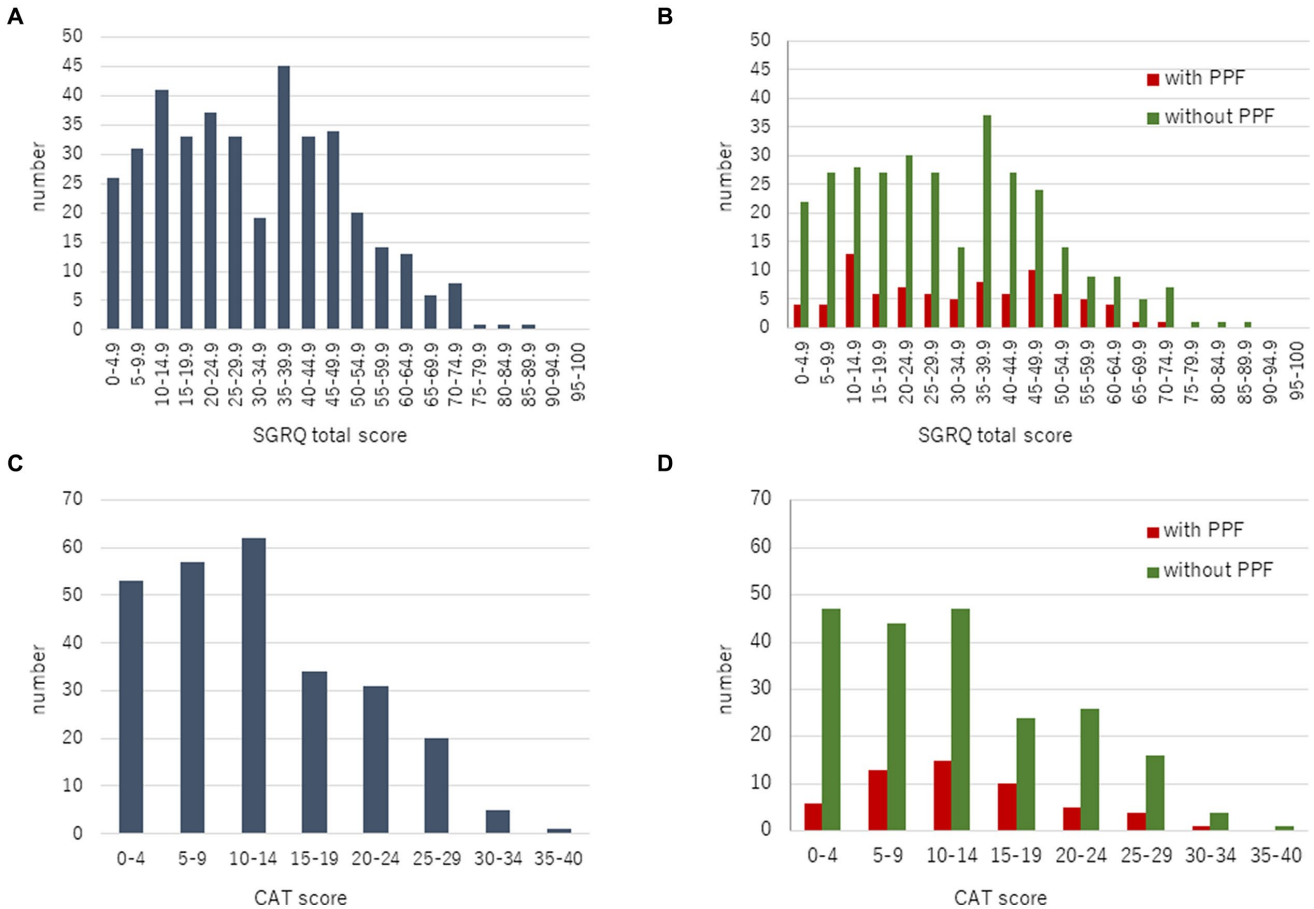
Distribution of the baseline SGRQ **(A)** and CAT **(C)** scores in all patients. Comparison of the baseline SGRQ **(B)** and CAT **(D)** scores between patients with and without PPF. CAT, COPD assessment test; PPF, progressive pulmonary fibrosis; SGRQ, St George’s respiratory questionnaire.

A total of 447 patients with non-IPF FILD were reviewed. Fifty-one were excluded due to death or lung transplantation within 24 months from the initial evaluation. Thus, 396 patients were included in the analysis set (Figure 1). Of these 396 patients, 19 had idiopathic non-specific interstitial pneumonia, 21 had fibrotic hypersensitivity pneumonitis, 163 had connective tissue disease-related ILD, six had idiopathic pleuroparenchymal fibroelastosis and 187 had unclassifiable ILD. Among 163 connective tissue disease-related ILD, 56 were rheumatoid arthritis, 38 were systemic sclerosis, 43 were myositis, 30 were sjögren syndrome, 8 were mixed connective tissue disease, and 4 were systemic lupus erythematosus (including overlap disease). Baseline characteristics are summarized in the Table 1. The median age was 65 years and 202 were male (51.0%). The median 6MWD was 560 meters. The median SGRQ and CAT scores were 29.6 and 11, respectively. Eighty-six (21.7%) showed PPF. Distribution of the baseline SGRQ and CAT scores are shown in Figure 2.

**Table 1:** Patients’ baseline characteristics.

	All patients^*^		PPF				
			yes (*n* = 86)		no (*n* = 310)	*p* value	
Age, year	65	(60–71)	67	(61–72)	65	(59–71)	0.07
Gender, male	202	[51]	43	[50]	159	[51]	0.90
FVC, %predicted	87.9	(73.5–103.6)	87.7	(74.0–102.3)	87.9	(73.1–103.8)	0.88
DL_CO_, %predicted	66.5	(52.1–82.5)	68.1	(53.3–84.4)	65.9	(51.7–81.3)	0.68
6MWD, m	560	(494–620)	559	(474–621)	563	(496–620)	0.55
SGRQ total	29.6	(15.0–44.9)	33.4	(15.6–47.5)	28.8	(14.9–42.8)	0.26
SGRQ symptom	37.3	(22.0–54.3)	38.1	(22.7–58.5)	36.8	(21.1–53.2)	0.37
SGRQ activity	36.8	(18.4–59.5)	39.7	(23.4–60.6)	36.5	(18.3–59.5)	0.31
SGRQ impact	21.1	(8.1–36.0)	25.6	(9.1–38.8)	20.1	(8.0–35.8)	0.43
CAT	11	(5–18)	11	(8–19)	11	(5–18)	0.38

Data are presented as median (interquartile range) or number [%]. CAT, COPD assessment test; DL_CO_, diffusing capacity of the lung for carbon monoxide; FVC, forced vital capacity; PPF, progressive pulmonary fibrosis; SGRQ, St George’s respiratory questionnaire; 6MWD, 6 min walk distance. ^*^N = 396 except for DL_CO_ (*n* = 384), 6MWD (*n* = 382), and CAT (*n* = 263).

### Changes in SGRQ

**Figure 3:**
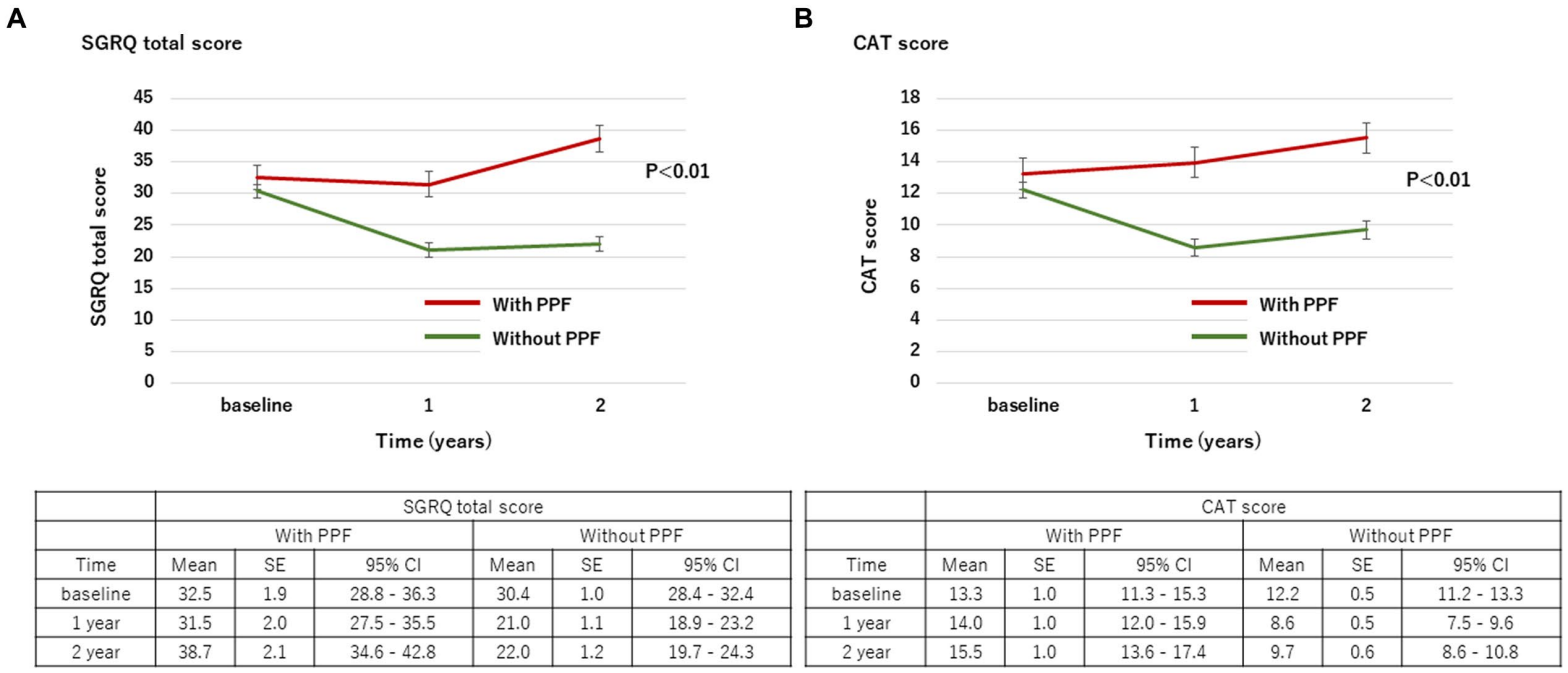
Change in the SGRQ total score **(A)** and CAT score **(B)** over 2 years from baseline. Standard error (SE) is derived from a mixed model for repeated measures. CAT, COPD assessment test; CI, confidence interval; PPF, progressive pulmonary fibrosis; SGRQ, St George’s respiratory questionnaire.

**Figure 4:**
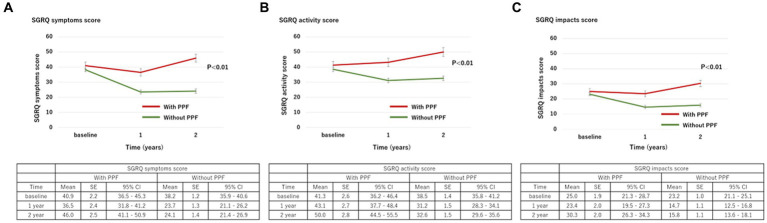
Change in each component of the SGRQ score (**(A)**, symptoms; **(B)**, activity; **(C)**, impacts) over 2 years from baseline. Standard error (SE) is derived from a mixed model for repeated measures. CI, confidence interval; PPF, progressive pulmonary fibrosis; SGRQ, St George’s respiratory questionnaire.

With regard to HRQoL, the mean change (standard deviation) in the SGRQ score over 2 years from the baseline was 5.8 ± 17.9 in patients with PPF and −9.5 ± 16.4 in those without. The SGRQ score was significantly higher in patients with PPF compared to those without (*p* < 0.01) (Figure 3A). Differences in each component of the SGRQ between patients with and without PPF are shown in Figure 4.

The difference between the baseline SGRQ score and the 2 year SGRQ score was evaluated in 262 patients. Among 86 patients with PPF who evaluated SGRQ at baseline, each number of patients who had evaluated the 2 year changes of SGRQ in each category of the criteria for PPF was 40 in 57 patients met the a relative decline of FVC ≥ 10%, 16 in 17 patients met a relative decline in FVC of ≥5–<10% with a relative decline in DL_CO_ of at least 15%, 15 in 16 patients met a relative decline in FVC of ≥5–<10% with increased fibrosis on high-resolution computed tomography, and 12 in 14 patients met a relative decline in FVC of ≥5–<10% with progressive symptoms.

**Figure 5:**
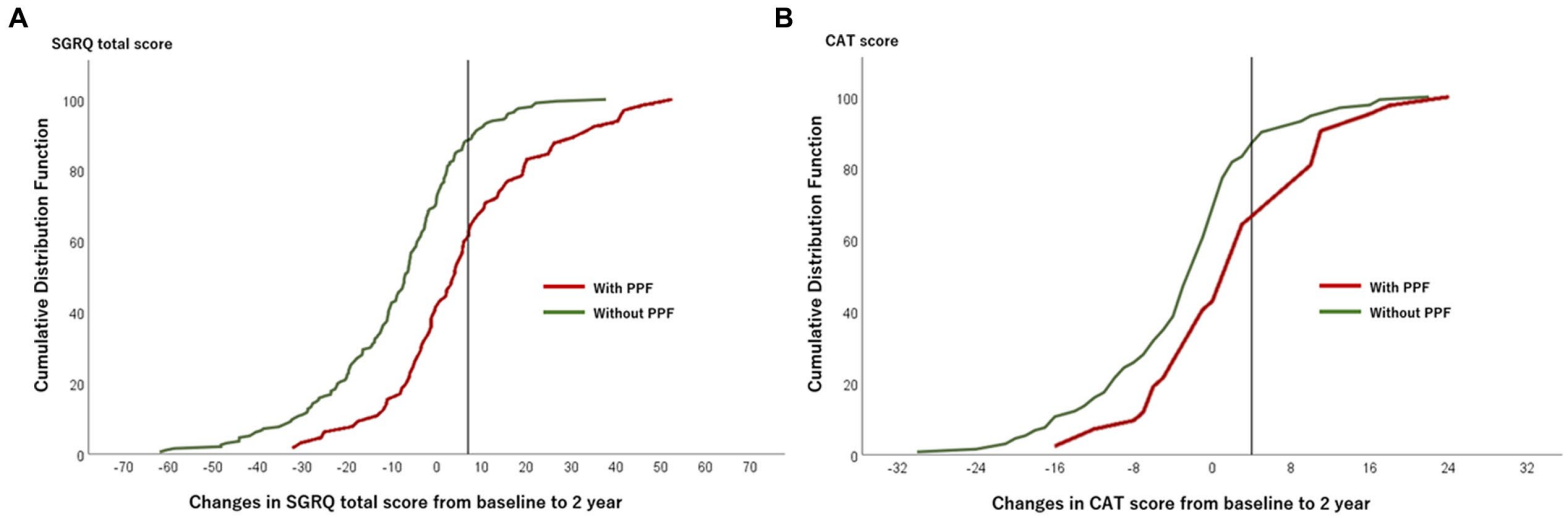
Plot of CDF for the SGRQ total score **(A)** and the CAT score **(B)** from baseline to 2 years in patients with and without PPF. The vertical lines show the threshold of the minimal clinically important difference (SGRQ total score, 7 points; CAT score, 4 points). CAT, COPD assessment test; CDF, cumulative distribution function; PPF, progressive pulmonary fibrosis; SGRQ, St George’s respiratory questionnaire.

**Figure 6:**
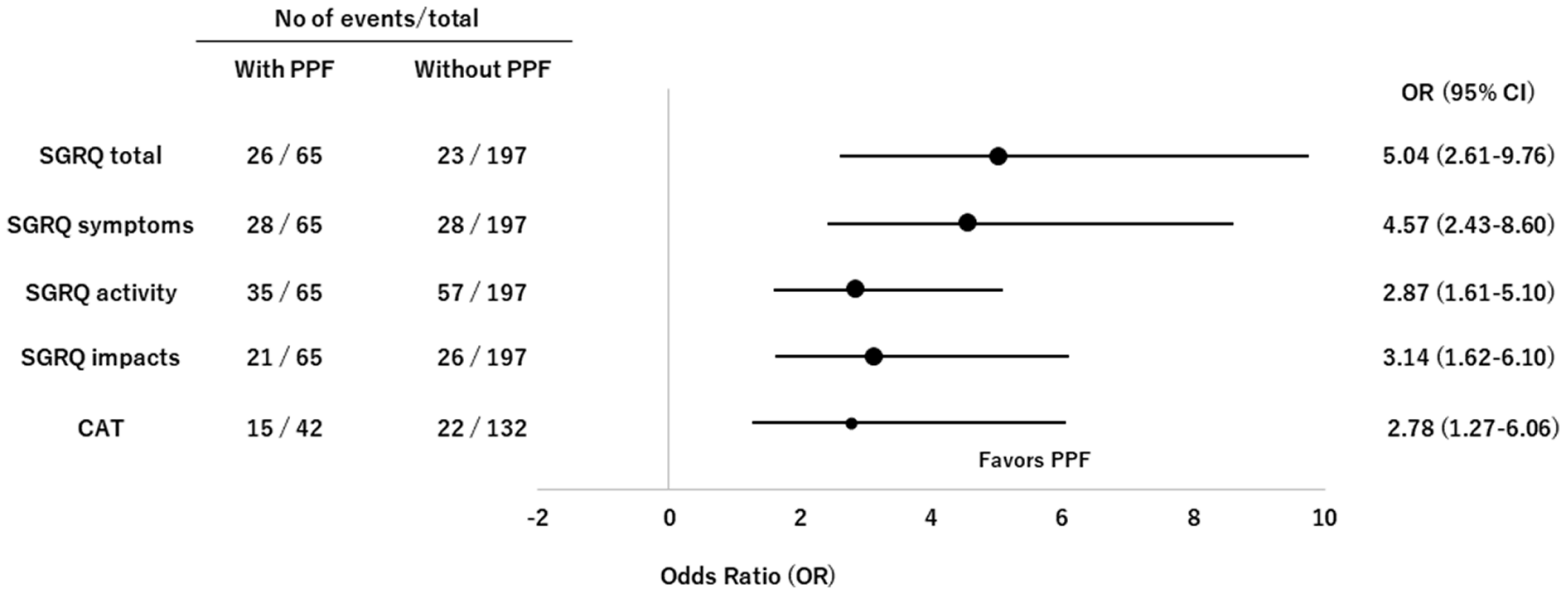
Forest plots of odds ratios for deterioration in the SGRQ and CAT scores in patients with non-IPF FILD. CAT, COPD assessment test; CI, confidence interval; FILD, fibrotic interstitial lung disease; IPF, idiopathic pulmonary fibrosis; OR, odds ratio; PPF, progressive pulmonary fibrosis; SGRQ, St George’s respiratory questionnaire.

The CDF plots provide a graphical presentation of the SGRQ change scores in patients with and without PPF (Figure 5A). Changes in the SGRQ total scores were significantly different between patients with and without PPF (*p* < 0.01). Clinically important deterioration over 2 years in the SGRQ total score was observed in 26 (40.0%) of 65 patients with PPF and 23 (11.7%) of 197 patients without PPF, respectively. PPF was significantly associated with clinically important deterioration in the SGRQ total score (odds ratio 5.04; 95%CI 2.61–9.76, *p* < 0.01) (Figure 6).

### Changes in CAT

The mean change (standard deviation) in the CAT score over 24 months from baseline was 2.0 ± 8.5 in patients with PPF and −3.1 ± 8.6 in those without. The CAT score was significantly higher in patients with PPF compared to those without (*p* < 0.01) (Figure 3B).

The difference between the baseline CAT score and the 2 year CAT score was evaluated in 174 patients. Among 54 patients with PPF who evaluated CAT score at baseline, each number of patients who had evaluated the 2 year changes of CAT score in each category of the criteria for PPF was 24 in 35 patients met the a relative decline of FVC ≥ 10%, 13 in 13 patients met a relative decline in FVC of ≥5–<10% with a relative decline in DL_CO_ of at least 15%, 9 in 9 patients met a relative decline in FVC of ≥5–<10% with increased fibrosis on high-resolution computed tomography, and 8 in 9 patients met a relative decline in FVC of ≥5–<10% with progressive symptoms.

The CDF plots provide a graphical presentation of the CAT change scores in patients with and without PPF (Figure 5B). Changes in the CAT scores were significantly different between patients with and without PPF (*p* < 0.01). Clinically important deterioration over 2 years in the CAT was observed in 15 (35.7%) of 42 patients with PPF and 22 (16.7%) of 132 patients without PPF, respectively. PPF was significantly associated with clinically important deterioration in the CAT score (odds ratio 2.78; 95%CI 1.27–6.06, *p* = 0.02) (Figure 6).

### Changes in exercise capacity

With regard to exercise capacity, the mean change (standard deviation) in the 6MWD over 24 months from baseline was −62.2 ± 120.7 in patients with PPF and 22.8 ± 75.6 in those without. The 6MWD was significantly lower in patients with PPF compared to those without (*p* < 0.01) (Supplementary Figure S1).

The CDF plots provide a graphical presentation of the 6MWD change scores in patients with and without PPF (Supplementary Figure S2). The difference between the baseline 6MWD and the 2 year 6MWD was evaluated in 252 patients. Clinically important deterioration over 2 years in the 6MWD was observed in 33 (52.4%) of 63 patients with PPF and 40 (21.2%) of 189 patients without PPF, respectively. PPF was significantly associated with clinically important deterioration in the 6MWD (odds ratio 4.10; 95%CI 2.24–7.51, *p* < 0.01).

## Discussion

We evaluated the changes in the SGRQ and CAT scores in patients with non-IPF FILD and PPF compared with those without PPF. Our data showed that both the SGRQ and CAT scores were significantly deteriorated in non-IPF FILD with PPF. The fact that up to 40% of patients with PPF had significant worsening of the SGRQ and CAT scores indicates that majority of patients with PPF do not experience a significant deterioration. On the other hand, only about 15% of patients without PPF had a significant deterioration of the SGRQ and CAT scores indicating that the HRQoL is unlikely to be worsened in patients without PPF. To our knowledge, this is the first study to assess the utility of the SGRQ and CAT scores in non-IPF FILD focused on PPF.

Our results showed that the mean change in the SGRQ total score from baseline to 2 years was about 6 points in patients with non-IPF FILD and PPF. A previous study (the INPULSIS trial) showed that patients with IPF had mean changes of about 4 points in the SGRQ total score in 52 weeks (20). Therefore, non-IPF FILD with PPF may have had a similar impact on the HRQoL to IPF.

The SGRQ is one of the most used tools for assessing HRQoL in patients with IPF (17, 21). Previous studies showed the SGRQ total score had a good correlation with FVC and was associated with prognosis in patients with IPF (21, 22). CAT score is also a valid HRQoL measurement and has a strong correlation with the SGRQ score in patients with IPF (23). The SGRQ and CAT scores have also been validated in patients with connective tissue disease-related ILD (18, 24, 25). Moreover, the CAT score was reported to be associated with poor prognosis in FILD (26). Although several questionnaires are available to evaluate HRQoL in patients with IPF and non-IPF FILD (27), little is known about their utility in patients with non-IPF FILD focused on PPF.

Our study showed that both SGRQ and CAT scores were significantly deteriorated in patients with non-IPF FILD and PPF. However, by using MCID to evaluate the deterioration of HRQoL, it was found that only about 40% of patients with PPF had detectable deterioration, while about 15% of patients without PPF had deterioration. Therefore, the current respiratory function test-based criteria for the progression of ILD has limited value in detecting deterioration in HRQoL. Considering that HRQoL affects prognosis independently of lung function (22, 26) and the criteria for PPF were defined from prognostic factors, HRQoL may be a good candidate for the criteria of PPF. Further studies are needed to determine whether HRQoL should be included in the criteria for PPF.

The present study showed that exercise capacity was also significantly deteriorated in PPF of ILD. Exercise capacity is reported to be a determinant of HRQoL in ILD and is a possible point of intervention. Several reports have shown that the improvement of 6MWD and HRQoL by pulmonary rehabilitation (28), while there are few studies focused on PPF of ILD, and it would be one of the future research topics.

This study has several limitations. First, it is a single-center study from a retrospective clinical cohort in Japan and the sample size for each type of non-IPF FILD was limited. There may be potential diagnostic bias and difficulty of evaluation in each type of non-IPF FILD because of the sample size and the variability of ILD diagnosis between countries. However, all diagnoses were confirmed by multidisciplinary discussion by ILD experts. Second, racial and ethnic differences may exist in patients’ perceptions. Prospective validation is needed to clarify these points. Third, it should be noted that we did not evaluate PPF according to the criteria proposed by the guideline in 2022 (1). Finally, we decided the threshold values of changes in the SGRQ and CAT scores and 6MWD based on previous studies (14–16). The optimal thresholds remain controversial and the thresholds applied in this study could have overestimated or underestimated the changes.

In conclusion, our results showed that the SGRQ and CAT scores were significantly deteriorated in patients with non-IPF FILD and PPF. Approximately 40% of patients with PPF experience significant deterioration of HRQoL, while those without PPF are less likely to experience deterioration of HRQoL. Our findings suggest that HRQoL may be a valuable tool for monitoring disease progression in non-IPF FILD patients with PPF, but the current criteria for the progression of non-IPF FILD has limited value in detecting the deterioration in HRQoL. We may need to consider an evaluation of HRQoL when assessing PPF in patients with non-IPF FILD.

## Data availability statement

The data that support the findings of this study are available from the corresponding author upon reasonable request.

## Ethics statement

The studies involving human participants were reviewed and approved by Tosei General Hospital. Written informed consent for participation was not required for this study in accordance with the national legislation and the institutional requirements.

## Author contributions

RT, TM, and YK designed the study, collected the data, and wrote the original manuscript. RT performed the data analysis. All authors contributed to the article and approved the submitted version.

## Conflict of interest

YK reports consultant and lecture fees from Boehringer Ingelheim Co., Ltd. and lecture fees from Sekisui Medical Co., Ltd. TF reports grants from Nippon Boehringer Ingelheim Co., Ltd., outside the submitted work. TJ reports lecture fees from Boehringer Ingelheim Co., Ltd., Astra Zeneca Co. Ltd., and Kyorin Co. Ltd.

The remaining authors declare that the research was conducted in the absence of any commercial or financial relationships that could be construed as a potential conflict of interest.

## Publisher’s note

All claims expressed in this article are solely those of the authors and do not necessarily represent those of their affiliated organizations, or those of the publisher, the editors and the reviewers. Any product that may be evaluated in this article, or claim that may be made by its manufacturer, is not guaranteed or endorsed by the publisher.
