# Supplementary material for: Changes in patient-reported outcomes in patients with non-idiopathic pulmonary fibrosis fibrotic interstitial lung disease and progressive pulmonary fibrosis
Source: Front Med (Lausanne). 2023 Jun 30;10:1067149. doi: 10.3389/fmed.2023.1067149 (PMC10347395; doi:10.3389/fmed.2023.1067149)
Supplement: Supplementary file 1 [file Image_1.PDF]

Supplementary Material

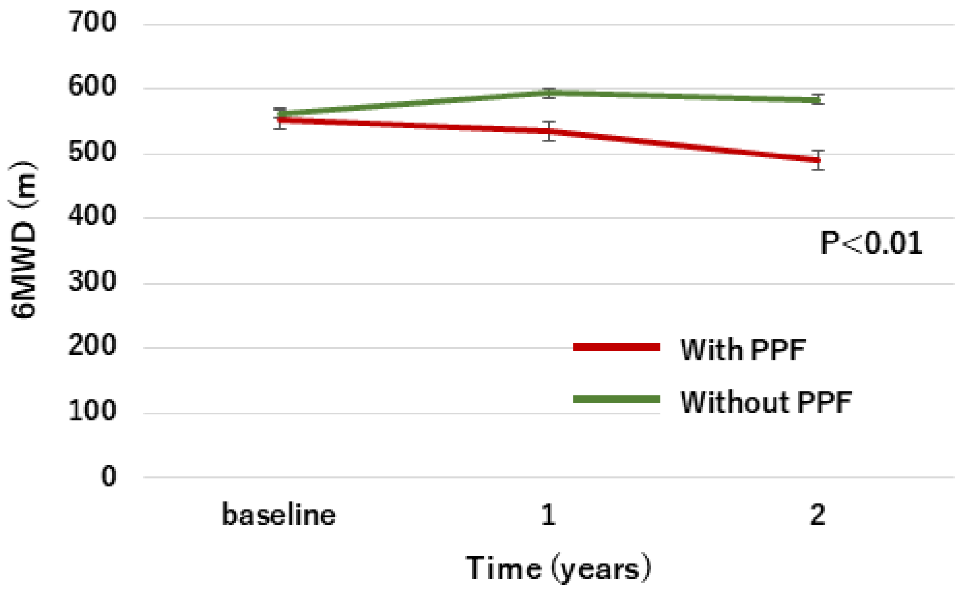

	6MWD (m)					
	With PPF			Without PPF		
Time	Mean	SE	95% CI	Mean	SE	95% CI
baseline	553.0	14.2	525.1 - 581.0	562.6	7.5	547.9 - 577.3
1 year	536.1	14.5	507.6 - 564.6	594.3	7.8	578.9 - 609.6
2 year	490.9	14.7	462.0 - 519.8	584.1	8.0	568.3 - 599.9

Supplementary Figure 1. Change in 6MWD over 2 years from baseline. Standard error (SE) is derived from a mixed model for repeated measures. CI, confidence interval; PPF, progressive pulmonary fibrosis; 6MWD, 6-min walk distance.

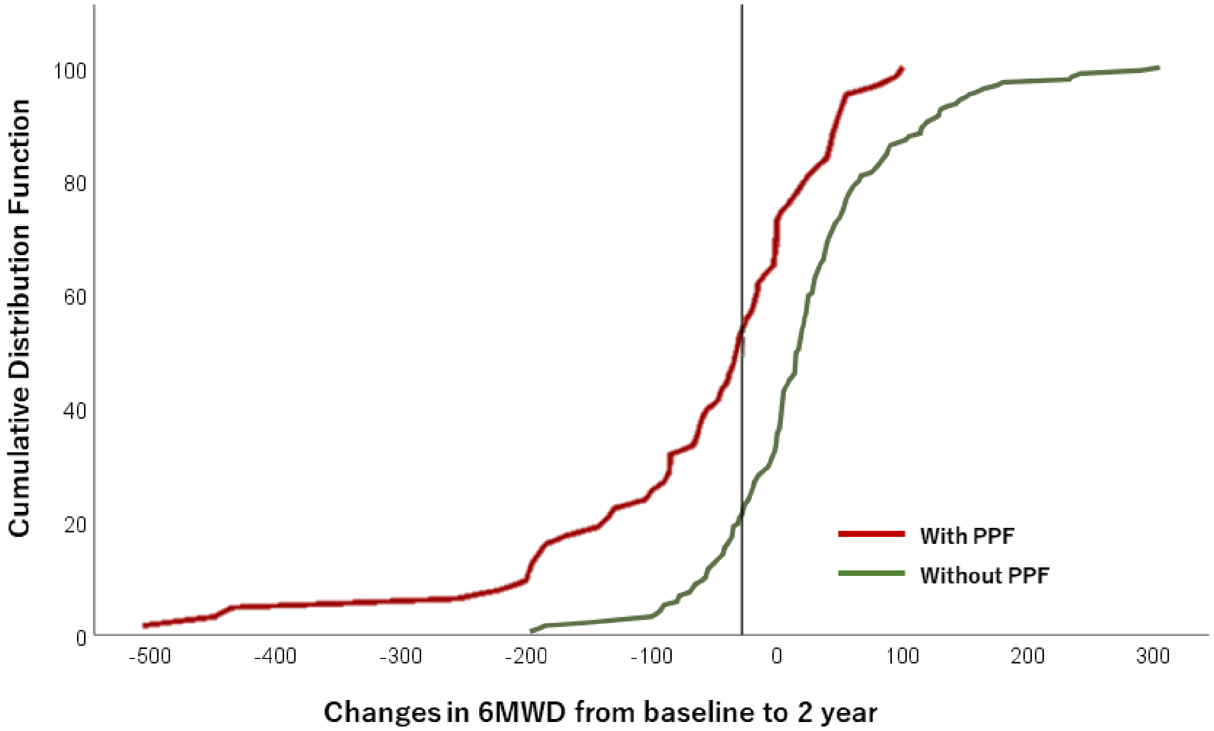

Supplementary Figure 2. Plot of CDF for the 6MWD from baseline to 2 years in patients with and without PPF. The vertical line shows the threshold of the minimal clinically important difference (6MWD, 28 m). CDF, cumulative distribution function; PPF, progressive pulmonary fibrosis; 6MWD, 6-min walk distance.
